# Threat to the French Swine Industry of African Swine Fever: Surveillance, Spread, and Control Perspectives

**DOI:** 10.3389/fvets.2019.00248

**Published:** 2019-07-29

**Authors:** Mathieu Andraud, Tariq Halasa, Anette Boklund, Nicolas Rose

**Affiliations:** ^1^Ploufragan-Plouzané Laboratory, Epidemiology Health and Welfare Department, ANSES, Ploufragan, France; ^2^Bretagne-Loire University, Rennes, France; ^3^Department of Veterinary and Animal Science, University of Copenhagen, Copenhagen, Denmark

**Keywords:** pig, notifiable disease, modeling, epidemiology, ASF

## Abstract

African swine fever (ASF) has one of the highest case-fatality rates among pig diseases. Europe was considered ASF-free for about two decades until 2007, when the virus was introduced into Georgia. Since then, it has been identified throughout Eastern Europe, and reached Belgium in late 2018, increasing the risk of ASF being introduced into neighboring countries—namely Germany, Luxembourg, the Netherlands, and France. French authorities have therefore reinforced surveillance measures to improve the probability of detecting ASF rapidly if it emerges in France. Predictive modeling may help to anticipate the extent of virus spread and evaluate the efficiency of these surveillance measures. A previously published and well-documented model that simulates ASF virus spread was therefore tailored to realistically represent the French situation in terms of the geographic distribution of swine production sites and the commercial trade between them on the one hand, and the implementation of surveillance protocols on the other. The outcomes confirmed the moderate spread of ASF through the swine trade network, a situation that had been previously highlighted for the case of Denmark. However, the diversity of the French pig production landscape has revealed a huge potential for the geographic dispersal of the virus, especially should the index case occur in a low-density area, with a median source-to-case distance reaching 300 km. Free-range herds, which are more likely to have interactions with wild boars, were also identified as potential entrance gate for the virus. Transmissions from conventional herds were quasi-exclusively due to swine movement on the commercial network, representing 99% of transmission events. In contrast, 81% of transmission events occurred in the neighborhood of the index herd when the virus was introduced in free-range herds. The current surveillance measures were found relatively efficient for detecting the virus in large herds, leading to detection rates of 94%. However, infections on smaller production sites—which often have free-range herds—were more difficult to detect and would require screening protocols specifically targeting these smaller herds.

## Introduction

African swine fever virus is a DNA virus belonging to the Asfarviridae family (1). With a case-fatality rate close to 100%, ASF has huge economic consequences not only at herd level but also at country level, due to the ban on exports following its emergence in a country officially free from the disease (2, 3).

To date, sub-Saharan Africa is seriously affected by ASF, the virus being endemic in several countries (4). Despite a few sporadic outbreaks successfully and rapidly contained and local endemicity on the island of Sardinia, Europe was considered free from ASF for about two decades until 2007, when the virus was introduced into Georgia via infected waste fed to domestic pigs. The waste originated from Africa and was brought to Georgia by boat (5). The virus spread through Russia and Ukraine, where it particularly affected wild fauna (6, 7). It continued to spread in Eastern Europe between 2012 and 2014, affecting both wild boars and domestic pigs. It spread very quickly through the Baltic countries (Latvia, Estonia, and Lithuania) before emerging in Poland (8) then finally reaching the Czech Republic in June 2017. Owing to strong control measures based on fencing in the infected area, intensive hunting of wild boars and the active search for carcasses, the Czech Republic was declared ASF-free by the European Commission in late February 2019, after 6 months without any ASF-positive cases. The opposite case is illustrated by Romania, where the first notification was reported in July 2017. This initial incursion had limited consequences in terms of local spread (9). However, the ongoing epidemic was initiated 1 year later, with more than 1,000 cases declared in October 2018 among both domestic and wild fauna. Apart from the Czech Republic, ASF is now considered endemic in affected Eastern European countries and a very real threat to Western Europe (10–12). In September 2018, the first notification of ASF cases in wild boars was reported in Belgium (13). Although the origin of the initial case has not yet been fully elucidated, a human role in this long-distance transmission seems the most likely (11, 14, 15). Since then and to date, ASF has continued to spread in wild boars in Belgium, despite the setting up of strict control measures with physical barriers, active searches for dead wild boars, the removal of carcasses from the environment, analysis of every culled or dead wild boar, and intensive hunting of wild boars within the reinforced observation area, similar to those implemented in the Czech Republic. To date (2019/03/26), 2059 wild boars have been tested in the surveillance zone, 708 of which have been found positive. The virus's incursion into France is imminent due to the shared boundaries between France and Belgium, the evidenced transmissibility of the virus, and the movements of wild boars. Although the introduction of ASF is more likely from this neighboring area via infected wild boars due to a continuum of the wild boar population across the border, a sporadic introduction into other areas should not be excluded because of the disease's increasing prevalence among both wild and domestic reservoirs in various Eastern European countries.

ASF is a notifiable disease according to European legislation, and strict containment and eradication policies are implemented in the European Union in the event of its suspicion. However, such policies require substantial human and financial resources, which drive the ability to detect the emergence of the virus in free countries and to control its spread in the meantime (hot period). Predictive modeling may therefore help to anticipate the extent of the virus's spread and to evaluate potential surveillance and control measures tailored to specific incursion scenarios. Halasa et al. recently developed such a model to analyze the epidemiological and economic consequences of ASF introduction on the swine production network in Denmark (16, 17). The modeling framework was adapted to reflect the real-life situation of the French swine production system and the implementation of recent surveillance and control policies adopted in response to the imminent ASF threat. The DTU-DADS-ASF model (16, 17) offers many different outputs directed toward epidemiology, economics, and resource requirements to be able to respond to the emergence of ASF in France. The objective of the present paper was to evaluate for the first time the epidemiological consequences of this virus's introduction into the French swine production system. We therefore essentially focused on epidemiological outcomes and detection procedures (e.g., the time to detection, duration of the epidemics, and the number of infected herds) to assess the efficiency of emergency surveillance plan in light of the diversity of the French swine production landscape.

## Materials and Methods

**Figure 1:**
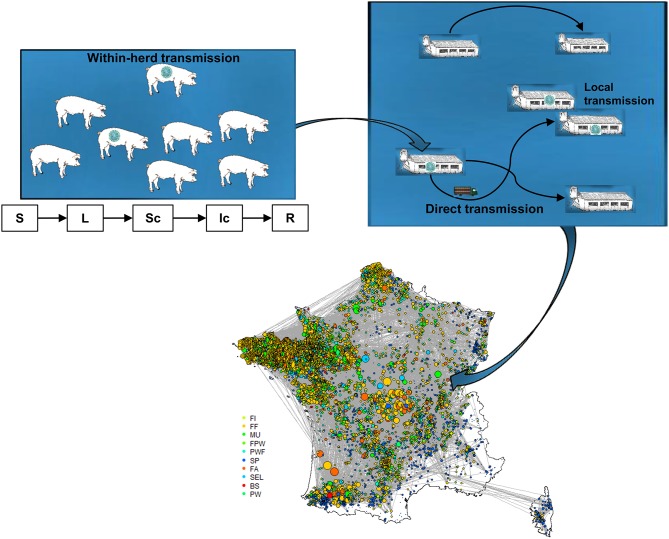
Schematic illustration of DTU-DADS-ASF epidemiological multiscale model (S, Susceptible; L, Latent; Sc, Subclinically infected; Ic, Clinically infected; R, Removed). Colors on map correspond to the different herd types considered (BS, boar studs; FA, farrowing; FF, farrow-to-finish; FI, finisher; FWP, Farrowing-post-weaning; MU, multiplier; PW, post-weaning; PWF, post-weaning—finishing; SEL, nucleus; SP, small production; TR, trade center; WB, wild boars) (18).

The modeling work consisted in tailoring the DTU-DADS-ASF model fully described in the literature by Halasa et al. (17). Only a brief overview of the hypotheses and modeling framework are therefore provided here; a schematic representation being illustrated in Figure 1. An illustrative example of data formatting is provided in Supplementary Material 1. The model— developed to analyze the consequences of ASF introduction in organized swine production systems—represents the course of global infection by coupling the dynamics of infection both within and between herds and representing official control measures.

### Within-Herd Transmission Model

The herd is considered as a homogenous, randomly-mixed population, independent of its physiological status. The pigs evolve through five health statuses: susceptible (*S*), exposed but not infectious (latent; *L*), not clinically affected but infectious (subclinical; *Sc*), clinically affected (clinical; *C*), and not playing a role in transmission (removed, *R*). Transmission is assumed to be either through direct contact between susceptible and infectious animals, or through environmental contamination due to infected leftovers. The environmental reservoir (*E*) is proportional to the number of ASF-related dead animals, and balanced by an exponential decay representing the virus's capacity for survival in the environment. Using the transmission rate (β) estimate of Guinat et al. (19), the force of infection exerted on susceptible animals at each time *t* (days) is expressed by

(1) $$\begin{array}{r} \lambda_{t}=\beta *\frac{\left(\mu *{Sc}_{t-1}\right)+\left(C_{t-1}\right)+\left(\varepsilon *E_{t-1}\right)}{N_{t-1}}\;, \end{array}$$

Where μ and ε are factors associated with the relative infectiousness of subclinical animals and infected leftovers, respectively; *N*_*t*−1_ represents the herd size at time *t* − 1. Transmission is governed by binomial processes with the infection probability at time *t* given by *p*_*t*_ = 1−*exp*(−λ_*t*_). The duration of the latent, subclinical and clinical stages of newly-infected animals are randomly drawn from probability distributions derived from Halasa et al. (20) (with a mean duration of 1, 2, and 5 days, respectively). The herd status is determined by the within-herd infection dynamics considering that at least one individual in the latent, subclinical or clinical stage makes the herd change to the corresponding status.

### Between-Herd Transmission Model

Although direct contact through animal movements is deemed to be essential for ASF transmission, the model framework accounts for four additional indirect transmission routes. The transit trucks used to move animals to the slaughterhouse may call in at several pig farms during the same round, and therefore represent a risk of ASF introduction. Indirect contact by external human interventions has been represented through two levels of risk linked to the frequency of contact with animals. Medium-risk contact represents the chance of ASF being introduced by veterinarians or rearing technicians who are frequently in contact with live animals, while a low-risk contact is related to sporadic visitors or feed trucks, which are potential vectors through environmental contamination. Finally, local spread was considered within a 2-km radius of an infected farm to represent potential transmission through shared material and fomites. For each transmission route, the probabilities of transmission between production sites are computed daily using a sequential approach.

#### Transmission by Direct Contact

For each infected farm, the number of outgoing animals potentially entering into direct contact with other animals is first considered based on the actual frequency of movements derived from the movement database (BDPorc). For direct contact modeling, the number of animals involved in the movement is next evaluated depending on the herd size and type of production site. The destination herd is then randomly selected based on (i) the distance between the seeder and receiving herd, (ii) the type of production site, and (iii) the herd size, which has to be large enough to receive the incoming animals. Finally, the probability of transmission is evaluated based on both the prevalence of infectious individuals on the seeder farm and the size of the incoming batch.

#### Transmission by Indirect Contact

For medium- and low-risk contact, the number of outgoing animals is again evaluated using a Poisson distribution with respective frequencies of contact derived from the literature (17, 21, 22). The force of infection exerted by an infected herd is proportional to the within-herd force of infection (Equation 1) with specific transmission rates (17, 23). The transmission probabilities also factor in the distance according to the same probability distribution as described by Halasa et al. (17).

Transmission caused by indirect contact through travel to the slaughterhouse is represented in the same way with the exception of the number of potential contacts, which follows an exponential distribution where the average path length (mean number of herds en route to the slaughterhouse) is used as the distribution mean (18).

Finally, to represent local spread due to shared material or fomites, all herds within a 2-km radius of an infected herd are subjected to a distance-based probability of infection related to the within–herd force of infection (Equation 1).

Different introduction scenarios were evaluated depending on the density zone and herd type of the initial case (nucleus/multiplier or randomly-selected herds). For each scenario in this study, 2,000 simulations were run to achieve convergence of the outputs with a stable mean behavior and variability. Equations and epidemiological parameters are detailed in the supplementary material of the original paper describing the modeling framework (17).

### Disease Detection and Control Measures

#### Passive Surveillance

In the context of the imminent threat of AFS being introduced into France, the surveillance measures described by Halasa et al. have been reinforced (24). Mortality scores are systematically recorded and analyzed over 14-day periods to monitor abnormal events. Two distinct strategies are considered depending on the herd size. A herd with over 1,000 pigs and twice the average mortality rate during the 14-day period—a rate set at 6% over the period of production (25)—is systematically subjected to a clinical inspection. In smaller herds (below 1,000 animals), clinical inspections are performed as soon as the morbidity (computed as the total number of both sick and dead animals) over two successive weeks reaches twice that of theoretical mortality. In the event of suspicion during a clinical visit, serological, and virological investigations are performed according to the sampling scheme described in Table 1. Blood and tissue samples are taken, prioritizing clinically-affected and dead animals. Whenever a suspicion is confirmed, stamping-out measures are immediately implemented and Surveillance and Protection Zones (SZ and PZ) are set up within a 10- and 3-km radius, respectively. The delay between confirmation and total stamping-out of the herd depends on the on-site culling capacity, set at 500 head per day nationwide based on expert opinions.

**Table 1:** Epidemiological predictions of simulated ASF epidemics with surveillance measures adopted in France (24).

	**Index cases**				
	**Nucleus/Multiplier/Farrowing herds**				**Free-range herds**
	**Zone 1**	**Zone 2**	**Zone 3**	**Zone 4**	
Proportion of undetected outbreaks (%)	12.6	7.1	6.7	6.4	84.8
Time to first detection (days)	15 [11; 21]	14 [11; 20]	14 [11; 20]	14 [11; 19]	9 [6; 26]
Duration of epidemics (days)	23 [15; 47]	22 [15; 38]	22 [15; 41]	21 [15; 36]	20 [6; 47]
Number of infected herds	2 [1; 8]	2 [1; 6]	2 [1; 6]	2 [1; 5]	1 [1; 4]
Number of detected herds	2 [1; 7]	2 [1; 5]	2 [1; 6]	2 [1; 5]	0 [0; 1]
Number of culled animals	3,680 [1020; 14293]	3,019 [1116; 9514]	3,121 [1074; 11498]	2,814 [1100; 9185]	0 [0; 1248]
Number of clinical visits	102 [20; 464]	96 [28; 366]	151 [38; 542]	235 [88; 633]	90 [0; 305]
Number of serological visits	6 [1; 73]	9 [1; 55]	18 [3; 95]	32 [7; 108]	0 [0; 13]
Number of virological visits	3 [1; 32]	3 [1; 18]	4 [1; 34]	8 [1; 36]	0 [0; 10]

**Figure 2:**
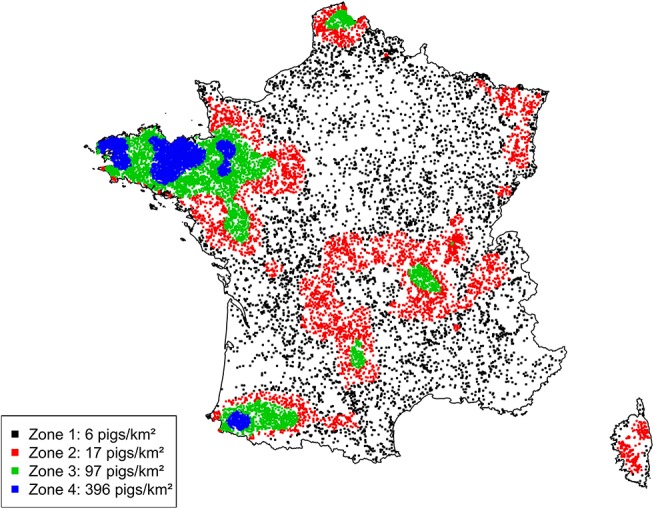
Geographic distribution of French swine production herds and definition of four density zones according to herd density quartiles.

*Index cases were selected according to the production type and density zones (Figure 2). Results are presented as medians and 5th and 95th percentiles*.

#### Surveillance and Protection Zones

All animal movements from and to these zones are prohibited for 45 (SZ) or 50 days (PZ), and all the herds in these two zones have to be monitored during that period. Clinical surveillance as described above is applied to both zones except for the second point: mortality notification is compulsory and systematically induces clinical inspection leading to further laboratory analyses (virology and serology). All herds in PZ are subject to mandatory serological surveillance visits, for which the sample size depends on the herd size and type (Table 1). Breeding animals on boar stud farms, in nucleus and in farrowing herds located in the surveillance zone are also systematically subjected to serological tests. All contacts are traced to identify the possible contact chains from and to infected herds over the 30 days prior to detection. Herds with epidemiological links to a suspected production site are subjected to clinical, serological and virological investigations.

### Data

The National Swine Identification Database (BDporc) has been registering all swine movements in France since 2010. Salines et al. (18) performed a social network analysis on movement data from 2012 to 2014 in which 10 types of production herds were identified. Data from year 2014, the latest full-year dataset at our disposal, were used to analyze the movement patterns between holding types. Two additional types of site were also considered: temporary stations (trade centers, TR) and slaughterhouses. A total of 21,446 sites were geographically located using the UTM-coordinate system so as to analyze swine movements based on distance. Four density zones were designed using Gaussian smoothing kernels, each containing 25% of the production sites. Movements were analyzed based on the “animal introduction model” (AIM) described by Salines et al. (18) to account for transmission through direct contact between animals from different production sites. Briefly, a one-mode directed network was built, wherein holdings were considered as nodes, and movements between two nodes were considered as links. All movements between two given holdings during the time period were aggregated into a single link. Links between holdings represented movements of animals being unloaded at farms. In-between movements forming a round were replaced by direct movements between holdings, i.e., intermediate transit movements of a truck through a farm without any animals being unloaded were excluded. In order to fulfill the model's input requirements, this network was further analyzed to characterize the connectivity and distance-related contact structure between the different types of production sites.

### Initialization and Analysis of ASF Spread

Based on pig trade network analysis, different scenarios regarding the type of herd in which ASF could be introduced were evaluated. We first considered introduction into breeding herds (nucleus, multiplier, and farrowing herds) having large outdegrees and movements mainly toward production herds (18). Seeder herds were randomly selected among the herds having at least three outgoing movements per month and herd sizes exceeding 360 animals (third and fourth quartiles). To analyze the impact of the geographic location of the seed herd and following the analysis by Halasa et al. (17), 2,000 repetitions were performed for the breeding herds in each density area. The second scenario considered the introduction of ASF into free-range production herds, which are potentially exposed to infectious pressure exerted by wild boar populations. Here the seeder herds were randomly selected regardless of their geographic locations. The latter scenario was run for 2,000 iterations to ensure full geographic coverage of mainland France and Corsica. The results are displayed in Table 1 and described in the next subsections.

## Results

### Geographic Analysis of Swine Movements

**Figure 3:**
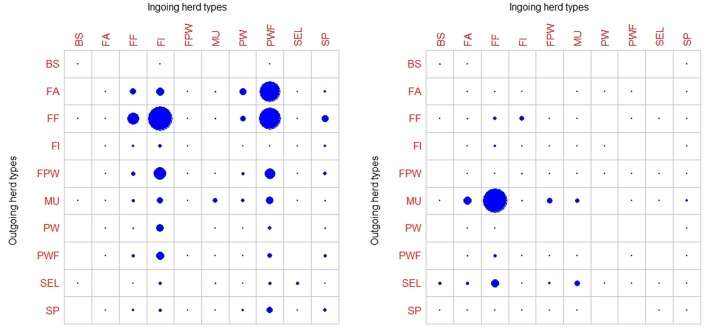
Characterization of commercial pig movements according to the different herd types sites (BS, boar studs; FA, farrowing; FF, farrow-to-finish; FI, finisher; FWP, Farrowing-post-weaning; MU, multiplier; PW, post-weaning; PWF, post-weaning—finishing; SEL, nucleus; SP, small production; TR, trade center; WB, wild boars) (18). Circle sizes represent the relative proportions movements of growing pigs (left) and breeding animals (right).

**Figure 4:**
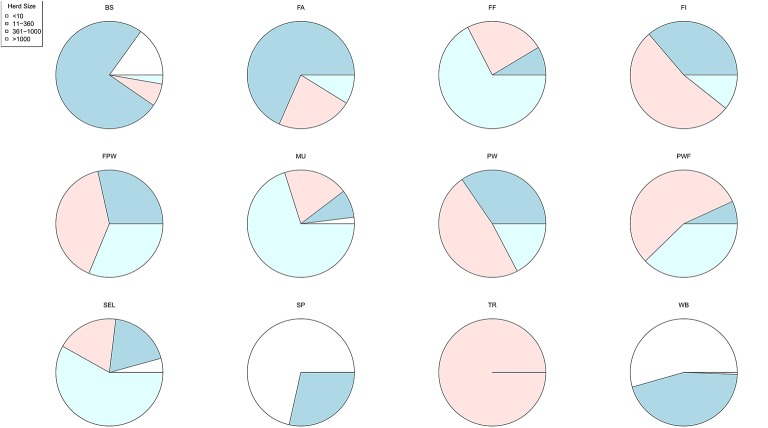
Distribution of herd sizes according to the type of production sites (BS, boar studs; FA, farrowing; FF, farrow-to-finish; FI, finisher; FWP, Farrowing-post-weaning; MU, multiplier; PW, post-weaning; PWF, post-weaning—finishing; SEL, nucleus; SP, small production; TR, trade center; WB, wild boars) (18).

**Figure 5:**
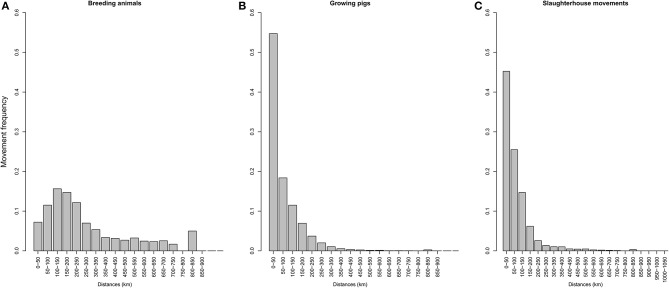
Distribution of distances involved in travel to the slaughterhouse depending on the type of production site: **(A)** growing pigs, **(B)** breeding sows. **(C)** represents the distribution of between-herd contact distances on the way to the slaughterhouse.

A large disparity in swine herd distribution was observed over continental France and Corsica, with swine densities varying from 6 to 396 pigs per km^2^ in the four zones. The highest densities were observed in the western and south-western parts of France (Figure 2). Each production site was assigned to its relative density zone to assess the impact of the index case location on ASF spread and control effectiveness. Excluding slaughterhouses, post-weaning-finishing and finishing herds have the highest indegree regarding growing pig movements, with 42 and 37% of between-site movements, respectively (Figure 3, left). In contrast, movements of breeding animals from multiplier herds were mostly directed toward farrow-to-finish pig herds (57% of movements) (Figure 3, right). Trade operators also received a high proportion of breeding animal movements (23%). However, these sites mostly represent a gathering step on the route to slaughterhouses or exportation; <1% of outgoing movements of breeding sows from trade centers were directed toward production sites. The herds were categorized according to their sizes, defined as the total number of sows, post-weaning and finishing pigs. Farrowing, farrow-to-finish, multiplier, and nucleus herds represent the largest proportion of large herds with 68, 67, 70, and 58% of these herds recorded as having more than 1,000 animals (Figure 4). Movements of pigs were reported at batch level, with batch size distribution depending both on the type and size of source herds. The average batch size contained 72 piglets but ranged between 1 and 432 animals (1–99% percentiles). The largest batch sizes were observed from farrowing and post-weaning herds, where batches could exceed 600 individuals. The distribution of the distances covered by breeding and growing animals revealed huge discrepancies (Figures 5A,B). The movements of growing pigs were short-ranged, 55% remaining within a 50-km radius, and the proportion of movements decreasing exponentially with increasing distance. In contrast, the farm-to-farm movements of breeding animals follow a long-tailed unimodal distribution pattern reaching a peak at 100–150 km distances but going as far as 850 km.

To evaluate the daily probability of occurrence of between-herd contact while traveling to the slaughterhouse—considered an epidemiological endpoint—movements were analyzed separately and taking into consideration their frequency for each herd type. Two production sites on average were visited on route to slaughterhouses. This value was used to evaluate the number of contacts drawn from an exponential distribution and balanced with the distance distribution between source herds and their associated slaughterhouses (Figure 5C).

### Epidemiological Consequences of ASF Introduction

#### Introduction Into Breeding Herds

**Figure 6:**
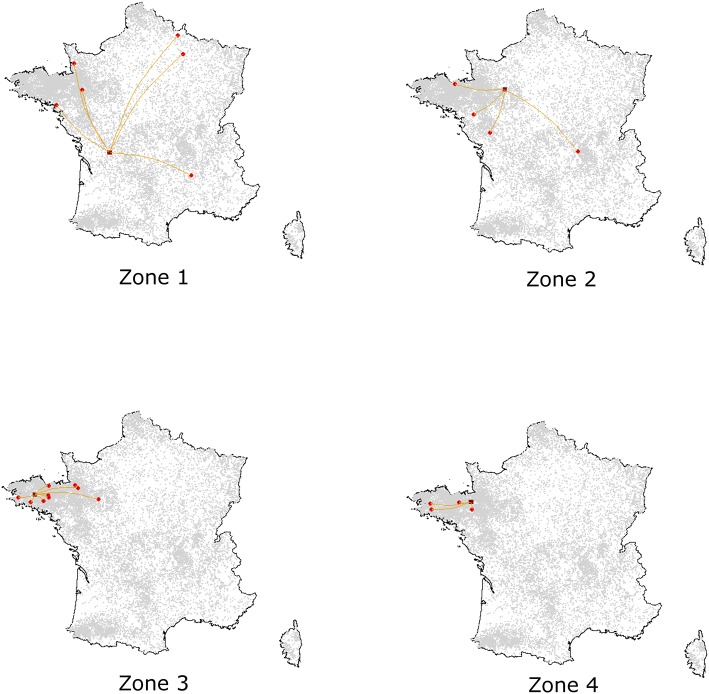
Illustration of simulated epidemics according to the density of the zone of introduction, assuming an introduction into Nucleus, Multiplier or Farrowing herds. For each introduction zone, one simulation was randomly selected among those with effective transmission events. Dark red squares represent the seeder herds while red spots correspond to the secondary outbreaks.

**Figure 7:**
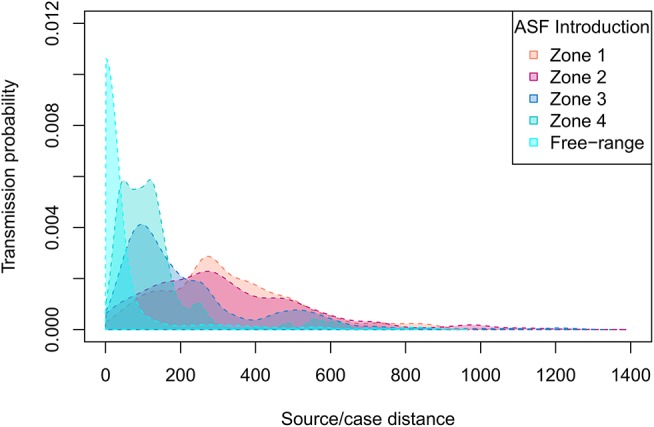
ASF source-to-case distance distributions depending on the density of the zone of introduction. Four density zones were defined according to the quartiles of swine production densities (Figure 2).

According to the model's outcomes, and applying the control and surveillance protocols described above, the spread of ASF among the swine production units in France was found to be relatively minor. An illustration of model behavior is provided in Figure 6. A median number of two herds were infected throughout the simulations whatever the region of introduction, and the number of infected herds remained below eight in 95% of the simulations. On average, 33% of the simulations did not lead to any secondary infections, the infection remaining limited to the seeder herd. The percentage of undetected herds was higher when the index case was introduced in low-density areas, reaching 12% (as opposed to 6–7% in other areas, Table 1) with long-distance transmission (Figure 7). Indeed, 75% of transmission occurrences remained in the introduction zone when the index case was located in high-density areas. This percentage fell to 40% in low-density areas and the infection spread up to 800 km away from the index location. Between 60 and 73% of transmission occurrences involved transmission from multiplier to farrow-to-finish herds. Fourteen per cent involved free-range herds when the index herd was in the lowest-density areas (zones 1 and 2), while this percentage fell to 6 and 2% when the virus was introduced into high-density areas (zones 3 and 4; data not shown).

Animal movements represented the major transmission route via the network, accounting for 99.4% of transmission occurrences; local transmission within a 2-km radius of infected herds accounted for 0.6% of transmission occurrences. Medium- and low-risk contacts were only sporadically involved throughout the simulation process (Table 2). The median time to first detection was evaluated as 15 days (14–21 days). The infection-to-detection time for secondary cases was shorter (11 days [3; 17]) due to the implementation of control measures after the initial detection. The average outbreak duration from introduction to disease extinction was evaluated at between 21 and 23 days, with variability ranging between 15 and 47 days. This rapid resolution was mainly achieved thanks to passive and active surveillance policies based on clinical inspections. The number of clinical, serological, and virological inspections was positively correlated with density in the area of the index case. Throughout the simulation process, the median numbers of clinical visits varied from 102 [20; 464] in low-density areas to 235 [88; 633] in high-density areas. However, the median number of serological investigations did not exceed 32 herds (high-density area), and only eight herds were subjected to virological investigations (ranges of 1–108 and 1–36, respectively, for serological and virological investigations at farm level).

**Table 2:** Proportion of ASF outbreaks caused by different mechanisms of disease spread in simulated epidemics in France.

	**Index cases**	
**Transmission routes (%)**	**Nucleus/Multiplier/Farrowing herds**	**Free-range herds**
Commercial movement of swine	99.36	11.79
Movement to slaughterhouse	0	0.17
Local contact	0.59	81.11
Low-risk contact	0	2.43
Medium-risk contact	0.05	4.5

#### Introduction Into Free-Range Herds

Transmission from index herds selected among free-range production sites was relatively rare. Only 12.5% of simulations revealed actual transmission from the index case to contact herds (87.5% with only one infected herd). When transmission occurred, the number of infected herds could reach 15, but 95% of these simulations resulted in the infection of eight herds or less. Sixty per cent of transmission occurrences involved small production herds with fewer than 80 pigs, and 37% concerned free-range herds. In contrast with previous results, local transmission was clearly identified as the major transmission route, accounting for 81% of events, whereas animal movements only represented 11% of transmissions (Table 2). Taking the whole simulation process, only 15.2% of infected herds were detected. Due to this low detection rate, which impairs the rapid implementation of control measures, and despite the low number of infected herds, the median epidemic duration was 20 days, though there was substantial variability ranging from 6 to 47 days. During this period, the number of clinical inspections reached 96 visits [1; 313] leading to a limited number of serological and virological investigations (4 [1; 36] and 1 [1; 11], respectively).

### Additional Screening of Small Herds (<300 Pigs)

The previous results clearly evidence the failure to detect infections occurring in small herds. We therefore assessed the impact of additional screening measures focusing specifically on these herds. We assumed that these herds were systematically subjected to clinical inspection as soon as they declared more than 15 dead or clinically-infected animals within two successive weeks (corresponding to an average morbidity of one animal per day), or when the whole population was affected during the same period. This latter assumption factored in the inspection of very small herds, which could have been easily omitted when considering only the previous surveillance measures.

The results obtained with this additional screening procedure are provided in Table 3. Whatever the location of the index herds (breeding herds), the required number of clinical, serological and virological inspections was only slightly increased, yet the detection rate increased drastically since only 1.2–1.8% of the infected herds remained undetected. The impact of the screening of small production herds was more pronounced when the infection was introduced into free-range herds. Indeed, the detection rate in this case also increased from 15 to 94%. Only 6% of the simulations resulted in secondary cases, still mainly due to the local transmission route. The time to first detection was similar to the previous scenario, but the median epidemic duration was limited to 12 days [12; 27]. Due to this rapid detection and short duration of the epidemics, the number of surveillance visits was significantly lower than in the baseline scenario, with only 32 [3; 192], 2 [1; 19], and 1 [1; 3] clinical, serological, and virological inspections, respectively.

**Table 3:** Epidemiological predictions of simulated ASF epidemics with surveillance measures adopted in France (7) and additional screening of small production sites.

	**Index cases**				
	**Nucleus/Multiplier/Farrowing herds**				**Free-range herds**
	**Zone 1**	**Zone 2**	**Zone 3**	**Zone 4**	
Proportion of undetected outbreaks (%)	1.7	1.3	1.4	1.4	3.7
First detection (days)	15 [11; 22]	14 [11; 21]	14 [11; 20]	14 [11; 19]	10 [1; 17]
Duration of epidemics (days)	23.5 [15; 50]	22 [15; 39]	22 [15; 44]	22 [14; 39]	12 [2; 27]
Number of infected herds	2 [1; 9]	2 [1; 6]	2 [1; 6]	2 [1; 5]	1 [1; 2]
Number of detected herds	2 [1; 9]	2 [1; 6]	2 [1; 6]	2 [1; 5]	1 [0; 2]
Number of culled animals	3,781 [1020; 15879]	3,048 [1110; 10406]	3,210 [1121; 13277]	2,985 [1100; 10187]	23 [0; 920]
Number of clinical visits	105 [21; 455]	106 [27; 358]	164 [42; 603]	255 [93; 646]	31 [3; 192]
Number of serological visits	7 [1; 83]	10 [1; 57]	19 [3; 108]	36 [8; 121]	2 [0; 19]
Number of virological visits	3 [1; 33]	3 [1; 21]	5 [1; 48]	11 [1; 49]	1 [0; 15]

*Index cases were selected according to the production type and density zones (Figure 2). Results are presented as medians and 5th and 95th percentiles*.

## Discussion

African swine fever has been spreading throughout Eastern Europe since 2007 and recently emerged in Western Europe (12). Although the threat has been highlighted for many years, the incursion of ASF into Belgium in September 2018 was relatively surprising since the closest infected countries were Poland and Hungary (13). The long-distance infectious process, although not fully elucidated, is deemed to be related to human activity. Since then, and despite strong control measures, ASF is continuing to spread among wild boars, with a recent report of more than 700 positive animals (26). Although fences have been set up along the neighboring boundaries, the risk of the ASF virus entering Luxembourg, Germany and France is non-negligible, and highlights the need for analytical tools to evaluate the economic and epidemiological consequences of such an introduction, as well as the efficiency and feasibility of surveillance/control policies in light of the local context and specificity of the population structure. The modeling framework designed by Halasa et al. (16) was dedicated to this issue and was used in their study to evaluate the spread of the virus among the Danish swine production network. This model was therefore chosen for application to the French situation, which required factoring in realistic geographic distribution of production sites as well as features related to the structure of French pig movements within the population (18). The authors have chosen not to discuss here the epidemiological assumptions and parameters behind the model, which were already well-discussed by the original authors. Therefore, the discussion will focus on the main conclusions and prospects for the future that could be derived from the model's outcomes. This study evaluated two different scenarios of introduction depending on the type of herd selected as the index case.

The first scenario considered an introduction into herds at the top of the pyramidal structure of the French swine production system: nucleus, multiplier and farrowing herds. Although these kinds of herds are expected to have the highest biosecurity measures, hence the lowest probability of virus introduction, the structural analysis of the pig trade network highlighted these herds as having the highest outdegrees and outgoing contact chains (18), so they may be considered the most vulnerable links in the production chain. In other words, these herds are likely to have epidemiological links with many commercial partners, which may lead to the transfer of pathogens whenever they become infected. Four density zones were designed to represent the quartiles of swine herd densities in France (6–396 pigs per square kilometer). Index herds were randomly selected within each zone to analyze the impact of their location on the epidemiological process and surveillance/control requirements. The zone of viral introduction was not found to have an impact on the number of cases, time to detection or the infection routes linked to the transmission process. The infection was transmitted relatively little, with a median of two infected herds [1; 5]. The transmission contact chains were limited to one main effector herd: seeder herds were the main transmission effectors, with secondary infected sources being only very sporadically involved (0.2–0.4% of simulations). This may be explained both by the pyramidal structure of the French pig production sector and by the reactiveness of the surveillance and control policies, with a median hot period evaluated at 15 days [11; 21]. However, further analysis revealed a huge difference in the geographic dispersion of the virus when index cases were simulated in low- or high-density areas, with distances between the sources and related cases potentially reaching 800 km when the index case was in a low-density area. This kind of transmission may allow the virus to emerge in more densely populated areas, in turn inducing a higher risk of local spread and potentially exposing a higher number of susceptible herds to infection, thus increasing the duration of epidemics nationally. Owing to the emergency surveillance and control policies set up in France following the emergence of ASF in Belgium, all large herds with twice the average mortality rates, and all smaller herds (fewer than 1,000 animals) with a 2-fold increase in morbidity (total number of clinically suspect and dead pigs) within a 14-day period have to be clinically inspected. This passive surveillance protocol was found to be relatively efficient in detecting ASF cases, with detection rates reaching 94%, while keeping the number of clinical visits to a minimum. Indeed, for outbreaks lasting between 15 and 41 days, the median number of clinical visits required was evaluated at 102 when the index case was in a low-density area, and up to 235 when in a high-density area, representing, respectively, 0.5 and 1% of the total number of production sites. A similar trend was obtained for serological and virological visits, which are mainly carried out to confirm clinical suspicions and which did not exceed 108 and 36, respectively, for high-density areas. However, it should be noted that the detection rate in the lowest-density area was lower than that obtained in the other areas (88 vs. 93–94%). This was mainly due to the failure to detect ASF in small herds, where the infection may fade out before any clinical visits could be planned.

The second scenario considered free-range pig herds as index cases, assuming this type of production site to be more susceptible to viral exposure through potential contact with wild boars. Keeping the same model structure and assumptions, the results obtained here were quite different from those obtained with the previous scenario. Indeed, the infection was contained within seeder herds in 87.5% of simulations. When transmission did occur, however, up to 15 herds could be infected, clearly identifying a real risk of transmission even from herds with little network connectivity. Furthermore, local transmission was clearly the main transmission route in this case, corresponding to transmission via material shared between neighboring herds, whereas pig movements played a major role in the previous scenario. Although the tracing of pig movements is well-documented (18) and may be used to identify epidemiological links between the different production sites, the local transmission pathway may be more difficult to assess due to the lack of traceability. This fact, along with the relatively small herd size of free-range sites, may contribute to the poor detection results achieved, with a detection rate of only 15.3% despite the global number of clinical, serological, and virological visits, which was similar to that obtained in low-density areas for the first scenario.

Based on these results, we decided to evaluate the impact of additional screening focusing on small herds with <300 animals. We therefore assumed a systematic clinical inspection of these small herds when either the number of clinically infected or dead animals exceeded the threshold value of 15 pigs over a 14-day period (on average more than one affected or dead pig per day), or the whole population was affected during the same period. With this additional surveillance measure, the detection rate exceeded 95% for all scenarios with the same number of visits. Although the hot periods were similar when the index case involved a free-range herd, the higher detection rate allowed a faster reaction time, thus avoiding further secondary cases (only 4% of simulations in this case were affected by secondary infections). Furthermore, in this specific case, shortening the infection process through enhanced screening of small herds also enabled the number of herd visits to be made during the epidemic to be reduced. These results clearly show the beneficial impact of targeted surveillance on small herds in addition to the emergency surveillance protocol already in place.

Although specific surveillance and control measures were integrated to represent the current situation in France, the model's outcomes were in close agreement with the results of Halasa et al. (16, 27). African swine fever undoubtedly has one of the highest case-fatality rates among pig diseases. However, our results evidenced a low degree of permeability of the swine production network regarding the virus's invasion potential, which is nevertheless highly dependent on the awareness and vigilance of all the actors and stakeholders in the production system (28) and the availability of the resources needed for clinical visits, sampling, analyses and on-site culling. Assumptions have been made in this evaluation, but the model could also be used to assess more precisely the impact in different scenarios of the availability of resources on the duration of ASF epidemics. In the present work, only domestic pigs were considered and a single introduction site was assumed. This major assumption may be overoptimistic regarding the pressure of infection exerted by wildlife in all the countries infected thus far (29, 30). Thulke and Lange developed a spatially explicit individual-based model to represent ASF spread in the wild boar population, and provided evaluations of control measures based on the implementation of fences, population management, and carcass removal procedures (31–33). Coupling these two approaches would provide the opportunity to analyze the infectious processes as a whole, which is a real challenge when attempting to manage a disease at the interface between wild and domestic ecosystems.

## Data Availability

The raw data supporting the conclusions of this manuscript will be made available by the authors, without undue reservation, to any qualified researcher.

## Author Contributions

TH developed the model. MA and NR designed the study and analyzed the results. MA performed data analysis, implemented the study, and wrote the manuscript. All the co-authors revised and approved the final manuscript.

### Conflict of Interest Statement

The authors declare that the research was conducted in the absence of any commercial or financial relationships that could be construed as a potential conflict of interest.

## Supplementary Material

Supplementary Material 1 Illustrative example of data formatting for feeding the DTU-DADS-ASF model. *rpert*(*n*, α, *m, M*) function generates *n* random numbers drawn from PERT distributions with parameters α, *m*, and *M* as the mode, the minimum and maximum values. More details can be found in Halasa et al. (17).

## References

[1] Galindo I Alonso C. African swine fever virus: a review. Viruses. (2017) 9:E103. 10.3390/v9050103 28489063 PMC5454416

[2] Gallardo C Soler A Nieto R Cano C Pelayo V Sanchez MA. Experimental infection of domestic pigs with african swine fever virus lithuania 2014 genotype II field isolate. Transbound Emerg Dis. (2017) 64:300–4. 10.1111/tbed.12346 25808027

[3] Guinat C Porphyre T Gogin A Dixon L Pfeiffer DU Gubbins S. Inferring within-herd transmission parameters for African swine fever virus using mortality data from outbreaks in the Russian Federation. Transbound Emerg Dis. (2018) 65:e264–71. 10.1111/tbed.12748 29120101 PMC5887875

[4] Arias M Jurado C Gallardo C Fernandez-Pinero J Sanchez-Vizcaino JM. Gaps in African swine fever: analysis and priorities. Transbound Emerg Dis. (2018) 65 (Suppl. 1):235–47. 10.1111/tbed.12695 28941208

[5] Rowlands RJ Michaud V Heath L Hutchings G Oura C Vosloo W. African swine fever virus isolate, Georgia, 2007. Emerg Infect Dis. (2008) 14:1870–4. 10.3201/eid1412.080591 19046509 PMC2634662

[6] Callaway E. Pig fever sweeps across Russia. Nature. (2012) 488:565–6. 10.1038/488565a 22932353

[7] Vergne T Korennoy F Combelles L Gogin A Pfeiffer DU. Modelling African swine fever presence and reported abundance in the Russian Federation using national surveillance data from 2007 to 2014. Spat Spatiotemporal Epidemiol. (2016) 19:70–7. 10.1016/j.sste.2016.06.002 27839582

[8] Smietanka K Wozniakowski G Kozak E Niemczuk K Fraczyk M Bocian L. African swine fever epidemic, Poland, 2014-2015. Emerg Infect Dis. (2016) 22:1201–7. 10.3201/eid2207.151708 27314611 PMC4918169

[9] Jurado C Martínez-Avilés M Torre AL Štukelj M Ferreira HCC Cerioli M. Relevant measures to prevent the spread of African Swine Fever in the European Union domestic pig sector. Front Vet Sci. (2018) 5:77. 10.3389/fvets.2018.00077 29713637 PMC5912175

[10] Mur L Martínez-López B Martínez-Avilés M Costard S Wieland B Pfeiffer DU. Quantitative risk assessment for the introduction of african swine fever virus into the European Union by legal import of live pigs. Transbound Emerg Dis. (2012) 59:134–44. 10.1111/j.1865-1682.2011.01253.x 21831148

[11] Mur L Martínez-López B Sánchez-Vizcaíno JM. Risk of African swine fever introduction into the European Union through transport-associated routes: returning trucks and waste from international ships and planes. BMC Vet Res. (2012) 8:149. 10.1186/1746-6148-8-149 22935221 PMC3485109

[12] Boklund A Cay B Depner K Földi Z Guberti V Masiulis M Epidemiological analyses of African swine fever in the European Union (November 2017 until November 2018). EFSA J. (2018) 16:5494 10.2903/j.efsa.2018.5494 PMC7009685 32625771

[13] Linden A Licoppe A Volpe R Paternostre J Lesenfants C Cassart D. Summer 2018: African swine fever virus hits north-western Europe. Transbound Emerg Dis. (2019) 66:54–5. 10.1111/tbed.13047 30383329

[14] Beltran-Alcrudo D Falco JR Raizman E Dietze K. Transboundary spread of pig diseases: the role of international trade and travel. BMC Vet Res. (2019) 15:64. 10.1186/s12917-019-1800-5 30795759 PMC6387505

[15] Chenais E Depner K Guberti V Dietze K Viltrop A Stahl K Epidemiological considerations on African swine fever in Europe 2014-201. Porcine Health Manag. (2019) 5:6 10.1186/s40813-018-0109-2 30637117 PMC6325717

[16] Halasa T Botner A Mortensen S Christensen H Toft N Boklund A. Control of African swine fever epidemics in industrialized swine populations. Vet Microbiol. (2016) 197:142–50. 10.1016/j.vetmic.2016.11.023 27938676

[17] Halasa T Bøtner A Mortensen S Christensen H Toft N Boklund A. Simulating the epidemiological and economic effects of an African swine fever epidemic in industrialized swine populations. Vet Microbiol. (2016) 193:7–16. 10.1016/j.vetmic.2016.08.004 27599924

[18] Salines M Andraud M Rose N. Pig movements in France: designing network models fitting the transmission route of pathogens. PLoS ONE. (2017) 12:e0185858. 10.1371/journal.pone.0185858 29049305 PMC5648108

[19] Guinat C Gubbins S Vergne T Gonzales JL Dixon L Pfeiffer DU Experimental pig-to-pig transmission dynamics for African swine fever virus, Georgia 2007/1 strain. Epidemiol Infect. (2016) 144:25–34. 10.1017/S0950268815000862 25989921 PMC4697298

[20] Halasa T Boklund A Bøtner A Toft N Thulke H-H. Simulation of spread of african swine fever, including the effects of residues from dead animals. Front Vet Sci. (2016) 3:6. 10.3389/fvets.2016.00006 26870740 PMC4735426

[21] Nielen M Jalvingh AW Horst HS Dijkhuizen AA Maurice H Schut BH Quantification of contacts between Dutch farms to assess the potential risk of foot-and-mouth disease spread. Prev Vet Med. (1996) 28:143–58. 10.1016/0167-5877(96)01042-2

[22] Boklund A Halasa T Christiansen LE Enoe C. Comparing control strategies against foot-and-mouth disease: will vaccination be cost-effective in Denmark? Prev Vet Med. (2013) 111:206–19. 10.1016/j.prevetmed.2013.05.008 23791121

[23] Nigsch A Costard S Jones BA Pfeiffer DU Wieland B. Stochastic spatio-temporal modelling of African swine fever spread in the European Union during the high risk period. Prev Vet Med. (2013) 108:262–75. 10.1016/j.prevetmed.2012.11.003 23419785

[24] DGAL/SDSPA/2019-41 Surveillance Événementielle et Gestion des Suspicions Cliniques de Pestes Porcines en Élevages de Suidés. (2019). Available online at: https://info.agriculture.gouv.fr/gedei/site/bo-agri/instruction-2019-41 (accessed April 04, 2019).

[25] IFIP Résultats des Élevages GTTT GTE – IFIP. Institut du Porc (2015-2016). Available online at: http://www.ifip.asso.fr/fr/resultats-economiques-gttt-graphique.html (accessed April 03, 2019).

[26] Portail de la Wallonie Mesures de Lutte Contre la Peste Porcine Africaine. (2019). Available online at: https://www.wallonie.be/fr/actualites/mesures-de-lutte-contre-la-peste-porcine-africaine (accessed April 04, 2019).

[27] Halasa T Bøtner A Mortensen S Christensen H Wulff SB Boklund A. Modeling the Effects of duration and size of the control zones on the consequences of a hypothetical African swine fever epidemic in denmark. Front Vet Sci. (2018) 5:49. 10.3389/fvets.2018.00049 29616228 PMC5868302

[28] Guinat C Wall B Dixon L Pfeiffer DU. English pig farmers' knowledge and behaviour towards African swine fever suspicion and reporting. PLoS ONE. (2016) 11:e0161431. 10.1371/journal.pone.0161431 27684556 PMC5042443

[29] More S Miranda MA Bicout D Bøtner A Butterworth A Calistri P African swine fever in wild boar. EFSA J. (2018) 16:e05344 10.2903/j.efsa.2018.5344 PMC7009363 32625980

[30] Podgorski T Smietanka K. Do wild-boar movements drive the spread of African Swine Fever. Transbound Emerg Dis. (2018) 65:1588–96. 10.1111/tbed.12910 29799177

[31] Lange M Alternative control strategies against ASF in wild boar populations. EFSA Support Publ. (2015) 12:843E 10.2903/sp.efsa.2015.EN-843

[32] Lange M Thulke H-H Elucidating transmission parameters of African swine fever through wild boar carcasses by combining spatio-temporal notification data and agent-based modelling. Stochastic Environ Res Risk Assess. (2017) 31:379–91. 10.1007/s00477-016-1358-8

[33] Thulke H-H Lange M Simulation-based investigation of ASF spread and control in wildlife without consideration of human non-compliance to biosecurity. EFSA Support Publ. (2017) 14:1312E 10.2903/sp.efsa.2017.EN-1312

